# Regulatory CD4+ T-Cell Subsets and Anti-Citrullinated Protein Antibody Repertoire: Potential Biomarkers for Arthritis Development in Seropositive Arthralgia Patients?

**DOI:** 10.1371/journal.pone.0162101

**Published:** 2016-09-01

**Authors:** Koen M. J. Janssen, Johanna Westra, Paulina Chalan, Annemieke M. H. Boots, Menke J. de Smit, Arie Jan van Winkelhoff, Arjan Vissink, Elisabeth Brouwer

**Affiliations:** 1 Department of Oral and Maxillofacial Surgery, University of Groningen and University Medical Center Groningen, Groningen, The Netherlands; 2 Department of Rheumatology and Clinical Immunology, University of Groningen and University Medical Center Groningen, Groningen, The Netherlands; 3 Center for Dentistry and Oral Hygiene, University of Groningen and University Medical Center Groningen, Groningen, The Netherlands; 4 Department of Medical Microbiology, University of Groningen and University Medical Center Groningen, Groningen, The Netherlands; JAPAN

## Abstract

**Objective:**

Seropositive arthralgia patients (SAP) are at high risk of developing rheumatoid arthritis (RA). This prospective study aimed to determine whether altered peripheral regulatory T-cells (Tregs) and defined subsets, besides a broadened anti-citrullinated protein antibody (ACPA) response, may qualify as biomarkers for RA development in SAP.

**Methods:**

Thirty-four consecutive SAP were prospectively assessed every 6 months for minimally 2 years. At inclusion, peripheral Treg (CD4^+^CD25^+^FoxP3^+^) numbers and subsets, defined as CD45RA^+^FoxP3^low^ naive Tregs (Fr I), CD45RA^-^FoxP3^high^ activated Tregs (Fr II) and CD45RA^-^FoxP3^low^ non-Tregs (Fr III), were compared to age- and sex-matched healthy controls (HC, n = 16) and treatment-naive RA patients (n = 12). SAP that developed RA were compared to non-switchers and analyzed for Treg numbers and Treg subsets at inclusion. Also, Treg numbers and subsets were compared in switched SAP before and at diagnosis. To assess the ACPA repertoire, IgG and IgA reactivity was measured against citrullinated peptides from fibrinogen, α-enolase and vimentin.

**Results:**

Treg numbers were similar between HC, SAP and RA patients. Although the bonafide Treg subsets Fr I and Fr II were comparable between groups, Fr III was increased in SAP compared to HC (p = 0.01). Fourteen (41%) SAP developed RA during follow-up. Their Treg numbers and subsets were comparable to non-switched SAP. At RA diagnosis, Treg numbers in switched SAP were similar to 6 months before. Switched SAP displayed a more diverse IgG ACPA repertoire compared to non-switched SAP (p = 0.046) and showed more IgA reactivity than non-switched SAP reaching significance for Fib1 only (p = 0.047).

**Conclusion:**

Numbers of Total Treg and bonafide Treg subsets are not indicative for RA development in SAP, opposed to the ACPA repertoire.

## Introduction

Rheumatoid arthritis (RA) is an autoimmune disorder characterized by chronic inflammation of the joints and by a breakdown of self-tolerance [1]. This breakdown is reflected by the induction of autoantibodies, such as rheumatoid factor (RF), anti-carbamylated protein antibodies (anti-CarP) and anti-citrullinated protein antibodies (ACPA) directed against self-proteins which are found in the majority of RA patients [2]. These autoantibodies can be detected in serum years before disease development [3,4]. Furthermore, levels of several cytokines, cytokine-related factors and chemokines congruent with an activated adaptive immune system are increased in the pre-clinical phase of RA [5].

In healthy conditions, the immunological equilibrium is maintained by the apoptosis of immature self-reactive lymphocytes in the thymus and activation-induced cell death of mature T-cells, as well as suppression of immune responses against self-proteins by regulatory CD4^+^ T-cells (Tregs) [6]. The function of Tregs is to suppress immune activation by modulating diverse cellular functions such as T-cell proliferation and cytokine production. Treg cells are defined by the expression of the nuclear transcription factor forkhead box protein 3 (FoxP3) and the expression of CD4 and CD25 on the cell-surface [7]. In RA patients, it remains unclear whether the frequency and/or the functionality of peripheral Treg cells is affected; some studies show a decrease [8,9], some report normal levels [10–14] whereas one study reports increased levels and enhanced activity in RA compared to healthy controls [15]. A subdivision of Treg cells into separate functionally different subpopulations was described by Miyara et al. [16], with functional subtypes such as CD45RA^+^FoxP3^low^ naive Treg cells (Fr I) which can convert to CD45RA^-^FoxP3^high^ activated Treg cells (Fr II) and non-functional CD45RA^-^FoxP3^low^ non-Treg cells (Fr III). Fr III cells, however, have no suppressive function, but are able to secrete cytokines such as IL-2, IFN-γ and IL-17.

The first aim of this study was to assess whether a change of regulatory T-cells (subsets) plays a role in the development of RA. We determined the numbers of the three aforementioned subpopulations of Treg cells in peripheral blood from seropositive arthralgia patients (SAP). We prospectively followed a group of SAP and assessed whether arthritis development was associated with changed numbers of regulatory T-cells and/or a shift in Treg subpopulations. By definition, SAP are seropositive for ACPA and/or IgM-RF and have (a history of) arthralgia but no arthritis. It has been reported that around 35% of these patients develop RA after a median follow up of 12 months [17]. The second aim of this study was to assess the extension of both the IgG and IgA ACPA repertoire in SAP. It has been shown that SAP with a more extended IgG ACPA repertoire have an increased risk of developing RA [18]. The IgA ACPA repertoire has been studied to a lesser extent than IgG ACPA, but the IgA ACPA repertoire might be of importance since mucosal inflammation has been hypothesized to play a role in the initiation of RA [19] and similar to IgG ACPA, IgA ACPA emerge before the onset of clinical RA [20].

## Materials and Methods

### Study subjects

Thirty-four consecutive SAP, seropositive for anti-CCP2 and/or RF (determined as described below) and having (a history of) arthralgia, but no arthritis were included. Patients were recruited at the rheumatology outpatient clinic of the University Medical Center Groningen between February 2012 and October 2013. The development of arthritis was confirmed by a senior rheumatologist after physical examination of 44 joints during half-yearly follow-up visits. Participants were instructed to contact the rheumatologist when they perceived progression of their joint complaints. Median follow-up time was 40 months (range 24–43 months). A group of 16 age and sex-matched healthy controls (HC) who were recruited among employees at the University Medical Center Groningen and subjects belonging to another healthy control group were included in the study [21]. Exclusion criteria for HC were inflammation, malignancy or use of immunosuppressive therapy. Furthermore, 12 consecutive treatment-naive newly diagnosed RA patients, diagnosed at the RA outpatient clinic of the UMCG between 2010–2011, fulfilling the ACR 1987 or 2010 criteria for RA, were included. These patients had their blood drawn at the moment of diagnosis before start of treatment with disease modifying anti rheumatic drugs (DMARD). Characteristics of patients and controls are described in Table 1.

**Table 1:** Clinical and serological characteristics at inclusion.

	Healthy controls(n = 16)	SAP(n = 34)	RA patients(n = 12)
Sex, n (% female)	12 (75)	24 (71)	8 (67)
Age, yr, median (IQR)	53 (50–59)	50 (38–56)	59 (52–70)*
SE status (% pos)^a^	62.5	69	67
CRP (mg/L), median (range)	NA	<5 (<5–29)	17 (<5–43)***
ESR (mm/h), median (IQR)	NA	11 (9–17)	28 (21–43)**
Anti-CCP2 positive, n (% pos)	NA	33 (97)	9 (75)
Anti-CCP2 (U/ml), median (IQR)	NA	99 (39–299)	240 (136–340)
RF positive, n (% pos)	NA	29 (85)	9 (75)
RF (IU/ml), median (IQR)	NA	54 (21–146)	346 (104–535)**
Lymphocyte count (10^6^/ml), median (range)^b^	1.85 (1.38–3.1)	2.05 (0.9–4.35)	1.90 (1.37–3.01)
CD4+ T-cell count (10^6^/ml), median (range)^b^	0.88 (0.46–1.57)	1.01 (0.37–2.44)	0.86 (0.64–1.51)
CD8+ T-cell count (10^6^/ml), median (range)^b^	0.38 (0.26–0.74)	0.36 (0.11–0.94)	0.36 (0.15–1.13)
CD19+ B-cell count (10^6^/ml), median (range)^b^	0.20 (0.14–0.48)	0.27 (0.05–0.87)	0.23 (0.08–0.42)
CD16+CD56+ NK-cell count (10^6^/ml), median (range)^b^	0.31 (0.15–0.58)**	0.21 (0.01–0.47)	0.24 (0.10–0.34)
DAS28-ESR, median (IQR)	NA	NA	5.67 (4.11–6.10)
TJC46, median (IQR)	NA	NA	8 (4–15)
SJC44, median (IQR)	NA	NA	7 (2–12)
VASgh, median (IQR)	NA	NA	60 (30–77)

SE = shared epitope (SE-containing alleles are HLA-DRB1*0401, *0404, *0405, *0408, *0101, *0102 and *1001); CRP = C-reactive protein; ESR = erythrocyte sedimentation rate; Anti-CCP2 = anti cyclic citrullinated peptides antibodies (positive score defined as > 10 IU/mL); RF = rheumatoid factor (positive score defined as ≥ 15 IU/mL); DAS28-ESR = disease activity score 28 using the ESR; TJC46 = tender joint count out of a possible 46; SJC44 = swollen joint count out of a possible 44; VASgh = visible analog scale for global health. NA: not applicable. Groups are compared to SAP (seropositive arthralgia patients).

^a^ Available for 16/16 HC, 33/34 SAP and 10/12 RA patients.

^b^ Available for 14/16 HC, 34/34 SAP and 10/12 RA patients.

*p<0.05

**p<0.01

***p<0.001.

Patients and controls gave their written informed consent at the start of the study, in compliance with the Helsinki Declaration. The study was conducted with the approval of the Medical Ethics Committee of the University Medical Center Groningen (METc 2009–118, 2011–306 and 2012–375).

### Flow cytometric analysis of regulatory T-cell subsets

Peripheral blood mononuclear cells (PBMCs) were isolated using Lymphoprep (Axis Shield, Oslo, Norway) centrifugation and stored in liquid nitrogen until further use. Cells were defrozen and subsequently fixed and permeabilized using the Foxp3 / Transcription Factor Staining Buffer Set (eBioscience, San Diego, CA, USA) and stained for flow cytometric analysis with anti-CD3-PerCP (BD Biosciences, San Jose, CA, USA), anti-CD8-APC-eFluor780 (eBioscience), anti-CD127-Alexa647 (Biolegend, San Diego, CA, USA), anti-CD25-PE (eBioscience), anti-CD45RO-Alexa700 (Biolegend), anti-CD45RA-Alexa605 (BD Biosciences) and anti-FoxP3-Alexa488 (eBioscience). Cells were measured on an LSR-II flow cytometer (BD Biosciences) and the data were analyzed with Kaluza 1.2 Analysis Software (Beckman Coulter, Woerden, The Netherlands).

Absolute numbers of lymphocytes in EDTA anti-coagulated blood were determined using the BD MultiTest TruCount method with sixcolor MultiTest reagents detecting CD45, CD3, CD4, CD8, CD19, CD16 and CD56 (BD Biosciences) according to the manufacturer’s instructions. Percentages of (functional) Treg subsets were characterized as described before [7,16] and CD4+ T-cell counts were used to calculate the absolute numbers of these subsets.

### Autoantibody measurements

Total Ig RF levels in serum were measured by turbidimetry with modular analyzer (Roche, Mannheim, Germany) with cut off level for RF-seropositivity ≥15 IU/ml. IgG anti-CCP2 levels were measured by fluorescent enzyme immuno assay on the Phadia 250 (Phadia Laboratory Systems, Phadia AB, Uppsala, Sweden) with cut off level for anti-CCP2 seropositivity ≥10 U/ml.

Specificity of the ACPA response was determined by testing reactivity against four well-known citrullinated peptides [18]; two peptides from fibrinogen (Fib1, β-chain amino acids 36–52, NEEGFFSACitGHRPLDKK and Fib2, β-chain amino acids 60–74, CitPAPPPISGGGYCitACit), one peptide from α-enolase (Eno1, KIHACitEIFDSCitGNPTVE) and one peptide from vimentin (Vim1, VYATCitSSAVCitLCitSSV). Citrulline-specific IgG and IgA reactivity was determined separately by measuring the difference in reactivity against the citrullinated and native form of the peptides, with the cut off defined as the difference in optical density (ΔOD) >2SD above the mean of 36 healthy controls, previously described [22].

### Statistics

Data were analyzed using GraphPad Prism 5 (Graphpad Software, San Diego, CA, USA). For group comparisons, a Mann–Whitney U test was used for continuous variables and Fisher’s exact test for categorical variables. A paired t-test was used for paired samples. Significance level α was 0.05.

## Results

### Circulating regulatory CD4^+^ T-cell numbers are not different between SAP and HC

**Fig 1:**
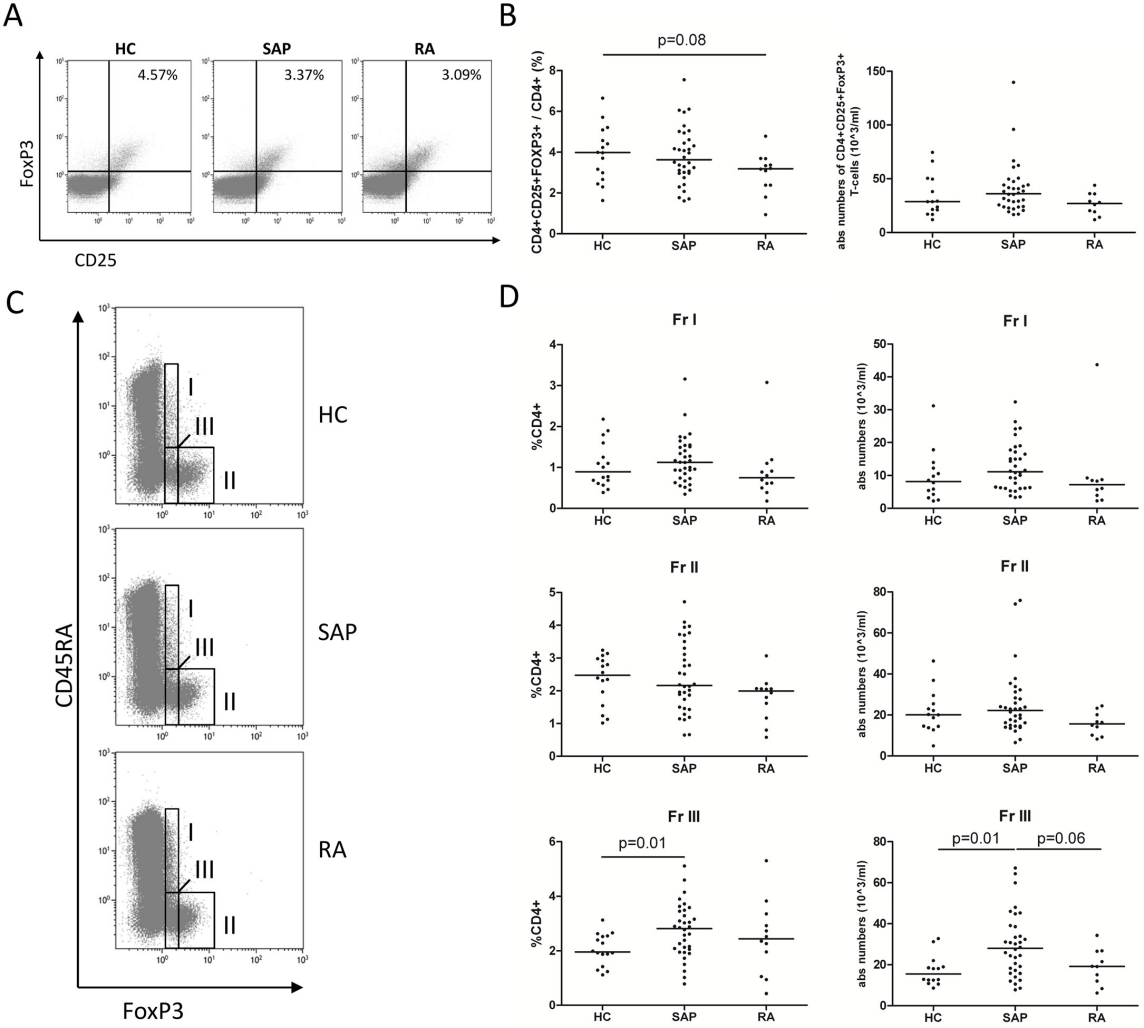
Analysis of Treg subpopulations in healthy controls (HC), seropositive arthralgia patients (SAP) at inclusion and treatment-naive rheumatoid arthritis patients (RA). (A) Three representative FACS analyses of CD25+FoxP3+ cells gated on CD4+ T-cells in HC, SAP and RA. (B) Percentages and absolute numbers of CD4+CD25+FOXP3+ cells negative for CD127 among CD4+ T-cells in HC, SAP and RA. (C) Three representative FACS analyses of Treg subpopulations gated on CD4+ T-cells in HC, SAP and RA. (D) Percentages and absolute numbers of naive Tregs (Fr I), activated Tregs (Fr II) and non-suppressive Tregs (Fr III) in HC, SAP and RA. Mann Whitney U test was used to compare groups. Horizontal bars indicate the median.

The frequency of CD4^+^CD25^+^FoxP3^+^ T-cells among PBMCs in HC, SAP at inclusion and RA was assessed by flow cytometry (Fig 1A). The frequency of CD4^+^CD25^+^FoxP3^+^ T-cells in SAP was comparable to HC and tended to be decreased in RA patients compared to HC (p = 0.08; Fig 1B). However, absolute numbers of CD4^+^CD25^+^FoxP3^+^ T-cells were comparable between HC, SAP and RA. Within total CD4+ Treg populations, the frequencies and numbers of Fr I, Fr II and Fr III Tregs defined based on the CD45RA and FoxP3 expression were assessed (Fig 1C). No differences between the groups were observed when comparing the two suppressive subtypes (Fr I and Fr II; Fig 1D), while an increase compared to HC was observed in both percentages and absolute numbers of Fr III for SAP (p = 0.01) but not for RA. In RA, absolute numbers of Fr III even tended to be decreased compared to SAP (p = 0.06).

### Peripheral Tregs in switched and non-switched SAP at inclusion

**Fig 2:**
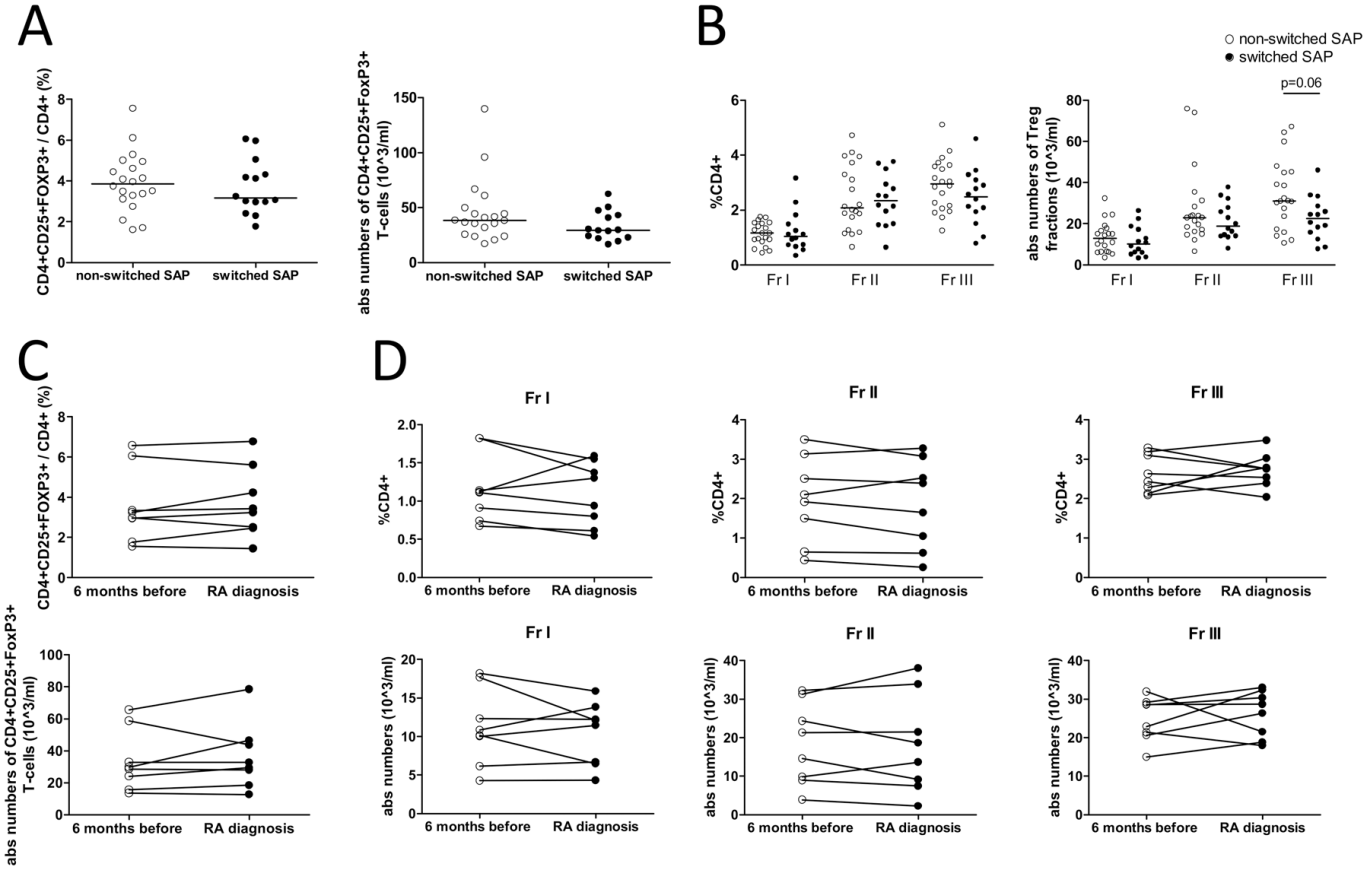
Treg subsets in SAP who switched or did not switch to RA. (A) Percentages and absolute numbers of CD4+CD25+FoxP3+ T-cells and (B) Treg subpopulations in non-switched vs switched SAP at inclusion. (C) Percentages and absolute numbers of CD4+CD25+FoxP3+ T-cells and (D) Treg subpopulations in switched SAP at the time of RA diagnosis and 6 months before. Mann Whitney U test was used to compare groups and paired t-test was used to compare paired samples. No significant differences were observed. Horizontal bars indicate the median.

Next, SAP that had not developed RA after a follow-up of at least 2 years were compared to the group of SAP that did develop RA during this period. Fourteen SAP (41%) developed RA after a median of 17 months (range 5–35 months). At the time of RA diagnosis, the median disease activity score 28 (DAS28) was 4.40 (IQR 3.78–5.24). Patient characteristics at inclusion of both groups and disease characteristics at time of RA diagnosis are given in Table 2. At inclusion, levels of CRP and ESR were comparable between non-switched and switched SAP. Although total lymphocyte counts were not different between non-switched and switched SAP, a trend for a decrease of CD4 T-cells was noted in the switched patients (p = 0.09). Yet, no significant differences between non-switched and switched SAP were observed in frequencies and absolute numbers of CD4^+^CD25^+^FoxP3^+^ Treg cells (Fig 2A). Likewise there were no significant differences between non-switched and switched SAP in frequencies and absolute numbers of Fr I and Fr II Treg subsets (Fig 2B). Although a tendency towards a decrease in absolute numbers of Fr III in switched SAP compared to non-switched SAP was observed (p = 0.06). The decrease of CD4 T-cells in switched SAP was not solely attributed to this decrease of Fr III, since CD4 T-cells levels without Fr III tended to be decreased too in switched SAP compared to non-switched SAP (p = 0.10).

**Table 2:** Patient characteristics at inclusion of SAP (seropositive arthralgia patients) subdivided for arthritis development and disease characteristics at RA diagnosis.

	Non-switched SAP (n = 20)	Switched SAP (n = 14)	*P*
Sex, n (% female)	15 (75)	9 (64)	0.70
Age, yr, median (IQR)	48 (38–53)	55 (37–66)	0.22
SE status (% pos)	63^a^	79	0.46
CRP (mg/L), median (range)	<5 (<5–29)	<5 (<5–19)	0.43
ESR (mm/h), median (IQR)	11 (9–16)	11.5 (10–22)	0.34
Anti-CCP2 positive, n (% pos)	19 (95)	14 (100)	1.00
Anti-CCP2 (U/ml), median (IQR)	90 (28–252)	211 (60–340)	0.14
RF positive, n (% pos)	16 (80)	13 (93)	0.38
RF (IU/ml), median (IQR)	39 (21–101)	120 (25–336)	0.10
Lymphocyte count (10^6^/ml), median (range)	2.15 (0.90–4.35)	1.96 (1.11–2.63)	0.28
CD4+ T-cell count (10^6^/ml), median (range)	1.15 (0.37–2.44)	0.98 (0.53–1.24)	0.09
CD8+ T-cell count (10^6^/ml), median (range)	0.36 (0.11–0.94)	0.37 (0.17–0.73)	0.74
CD19+ B-cell count (10^6^/ml), median (range)	0.27 (0.07–0.87)	0.27 (0.05–0.49)	0.89
CD16+CD56+ NK-cell count (10^6^/ml), median (range)	0.20 (0.07–0.46)	0.21 (0.01–0.47)	0.90
Disease characteristics at RA diagnosis after 5–35 months of follow-up			
DAS28-ESR, median (IQR)	NA	4.40 (3.78–5.24)	–
CRP (mg/L), median (range)	NA	10 (<5–36)	–
ESR (mm/h), median (IQR)	NA	19 (13–36)	–
TJC46, median (IQR)	NA	7 (2–12)	–
SJC44, median (IQR)	NA	3 (2–8)	–
VASgh, median (IQR)	NA	70 (30–78.5)	–

SE = shared epitope (SE-containing alleles are HLA-DRB1*0401, *0404, *0405, *0408, *0101, *0102 and *1001); CRP = C-reactive protein; ESR = erythrocyte sedimentation rate; Anti-CCP2 = anti cyclic citrullinated peptides antibodies (positive score defined as > 10 IU/mL); RF = rheumatoid factor (positive score defined as ≥ 15 IU/mL); DAS28-ESR = disease activity score 28 using the ESR; TJC46 = tender joint count out of a possible 46; SJC44 = swollen joint count out of a possible 44; VASgh = visible analog scale for global health NA: not applicable.

^a^ Available for 19/20 non-switched SAP.

### Treg proportions do not alter shortly before switch to RA

A shift in Treg numbers might occur shortly before the switch to RA and be indicative for RA development. Therefore, the Treg levels in the eight SAP of whom PBMCs were available, were compared at the time of RA diagnosis to six months before. Again, frequencies and absolute numbers of CD4^+^CD25^+^FoxP3^+^ Treg cells (Fig 2C) and Treg subsets (Fig 2D) were not affected by the switch to RA. However, when compared to inclusion, CRP and ESR levels tended to be increased six months before RA diagnosis (p = 0.06 and p = 0.09, respectively) and were significantly increased at the time of RA diagnosis (p = 0.03 and p = 0.02, respectively).

### ACPA repertoire in serum

**Fig 3:**
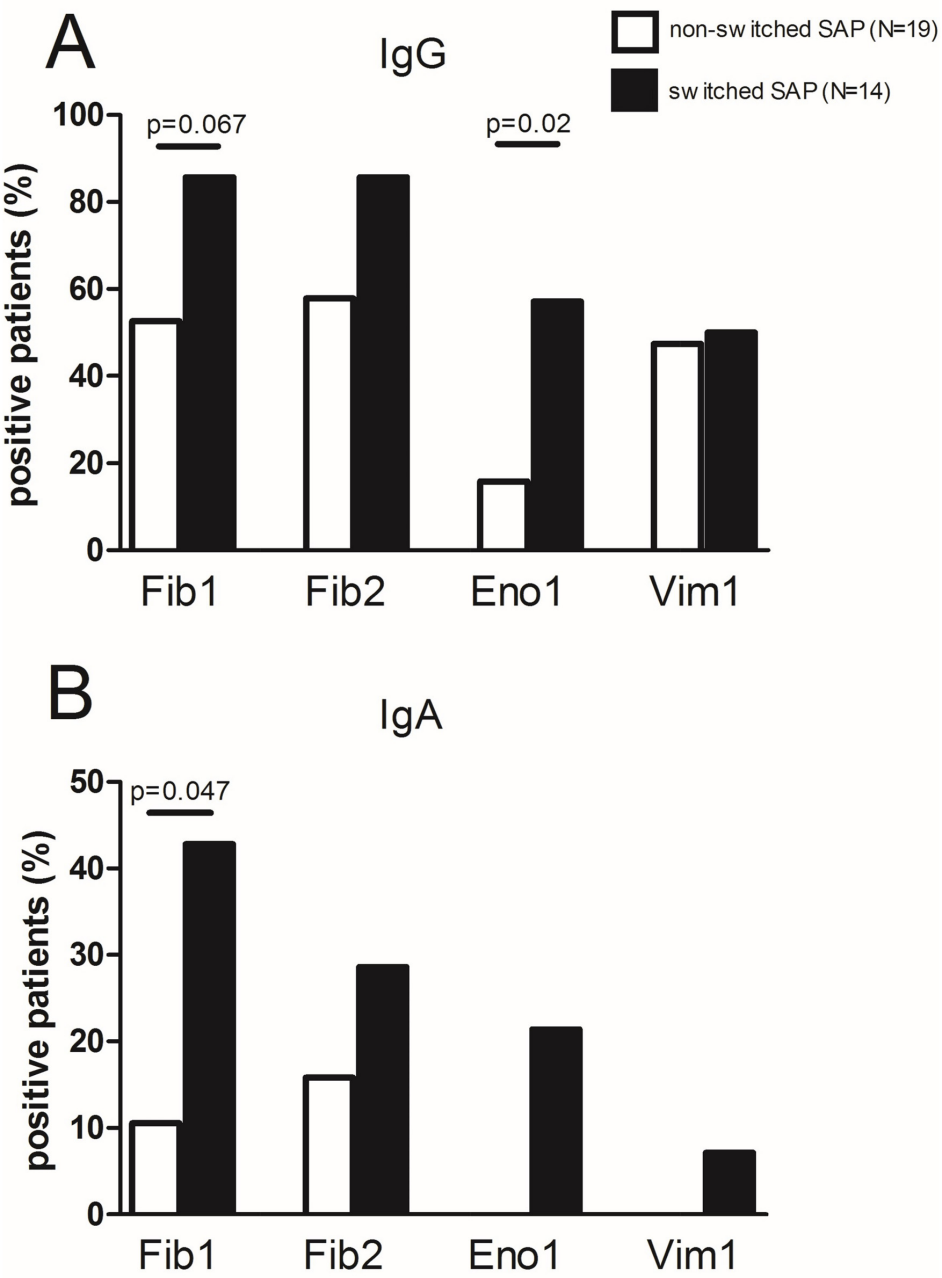
Reactivity against citrullinated peptides from fibrinogen (Fib1, Fib2), α-enolase (Eno1) and vimentin (Vim1) in SAP sera at inclusion. (A) IgG seropositivity (B) IgA seropositivity. Reactivity is shown as percentage within the group of patients that switched or did not switch to RA. Fisher’s exact test was used to compare groups.

Anti-CCP2 and RF levels were not significantly increased at the time of diagnosis compared to six months before diagnosis (S1A and S1B Fig). Since almost all our SAP were anti-CCP2 positive, it was of interest whether the switched SAP displayed a more widespread ACPA repertoire compared to non-switched SAP. Therefore, reactivity was measured against 4 different citrullinated peptides. Regarding IgG ACPA, switched SAP recognized significantly (p = 0.046) more peptides at inclusion (median 3, IQR 2–4) compared to non-switched SAP (median 2, IQR 0–3). The number of peptides recognized by switched and non-switched SAP remained stable during follow-up (data not shown). Fr III includes effector T-cell subsets such as Th1 and Th17, cells which might migrate into inflamed synovia. Anti-CCP2 titers and IgG ACPA repertoire did not correlate with absolute numbers of Fr III (S1C and S1D Fig), however. All 4 citrullinated peptides were more often recognized by switched SAP compared to non-switched SAP reaching only significance for Eno1 (p = 0.02, Fig 3A). As expected, IgA reactivity against the citrullinated peptides was less often observed in SAP compared to IgG reactivity. However, switched SAP showed more IgA reactivity than non-switched SAP reaching significance for Fib1 only (p = 0.047, Fig 3B). Furthermore, IgA ACPA positive patients displayed a broad IgG ACPA peptide repertoire (median 3 peptides, range 2–4). In switched SAP 43% (n = 6/14) of the switched patients showed IgA reactivity against at least 1 citrullinated peptide while in non-switched SAP this was only 20% (4/20).

## Discussion

Early diagnosis and treatment of RA is of paramount importance for achieving a better disease outcome [23]. Therefore, it is necessary to understand which immunological factors play a role in the progression of a SAP towards RA since this could help to identify individuals who are at “high risk” of developing RA [24,25]. The current study assessed whether peripheral CD4^+^CD25^+^FoxP3^+^ Treg numbers are changed in SAP and whether these changes are predictive for RA development. Like other studies in diagnosed RA patients [8,9,26,27], the current study also pointed towards a decrease in percentages of peripheral regulatory T-cells in RA.

When looking into detail to the Treg subsets of the studied groups, an increase of CD45RA^-^FoxP3^low^ non-Treg cells (Fr III) was observed in SAP compared to HC. Fr III does not exert a suppressive function, but is capable of secreting IL-17 [16]. Thus, the increase in Fr III might be attributed to a shift in the Th17/Treg balance as has been reported for (treated) RA patients [26]. However, in our study, no increase of Fr III in RA patients was observed. Rather, the Fr III subset tended to decrease in RA patients compared to SAP. The latter is in line with previous work from our group indicating that peripheral CD4+CD161+ T cells (Th17 lineage cells) are increased in SAP, but decreased in RA patients at disease onset with an enrichment of these cells in the joints [28]. The tendency that peripheral Fr III numbers are lower at inclusion in switched SAP compared to non-switched SAP might indicate that Th17-cells migrate to inflammatory sites in the joints, although an earlier immunohistochemical study could not detect an increase of T-cells in synovial biopsies from SAP who later developed RA compared to HC [29]. Another study in RA patients revealed a decrease in peripheral CD45RA^-^FoxP3^high^ activated Treg cells (Fr II) compared to HC [30], but that study assessed Fr II in (longstanding) RA-patients who were mainly treated with DMARDs and anti-TNF-α and not treatment-naive RA patients as assessed in our study.

The results of our study showed that there is no change in peripheral Treg and bonafide Treg subsets in the 6 months prior to arthritis diagnosis. In contrast to a recent study by Hunt et al. [31] who studied several T-cell subsets in a larger cohort of comparable patients, we do not confirm an additional value of decreased Treg frequencies for predicting RA development. A difference in follow-up periods between the studies may underlie this discrepancy. Hunt et al. [31] assessed SAP every 3 months in the first year after inclusion and only on clinical indication thereafter. That approach is significantly different from our approach assessing SAP every 6 months until they switched to RA. Interestingly, Hunt et al. [31] showed that a number of SAP who developed arthritis switched from normal frequencies to abnormal decreased peripheral frequencies of Treg or naive T-cells or increased proportions of inflammation related T-cells in the period before arthritis development compared to non-switched patients. Unfortunately, Hunt et al. [31] did not specify how many of the switched patients shifted towards decreased Treg frequencies and at which time point before arthritis development this switch had occurred. The omission of these data makes it difficult to appreciate and explain the discrepancies in results between Hunt et al. [31] and our study.

Our study showed that SAP who later progressed to RA recognized more citrullinated peptides than SAP who did not switch to RA during follow-up. Broader reactivity was observed for both IgG and IgA ACPA in switched SAP. IgG ACPA epitope spreading has been implicated in arthritis development of SAP also by others (18), however, to the best of our knowledge, involvement of IgA ACPA epitope spreading in SAP progression to RA has not been demonstrated yet. Therefore, it remains of importance to prospectively follow IgA ACPA positive non-switched SAP for the development of RA in future studies. Comparable to Treg numbers, serum levels and epitope spreading of autoantibodies remained unchanged at RA development compared to 6 months before as also was reported by others [18]. Apparently, SAP represent a subgroup of patients in which ACPA epitope spreading happens before the start of joint complaints but not before RA development, while another study in pre-symptomatic RA patients reports an expansion of the ACPA repertoire in the year before the development of RA [32].

Tregs are capable to suppress the emergence of long-lived plasma cells that produce autoantibodies in RA [33]. Animal experiments revealed that CD25+ cell-depleted DBA/1 mice had more severe disease and higher autoantibody levels than control mice with collagen induced arthritis (CIA) [34]. We did not find a negative correlation between Treg numbers and anti-CCP2 levels in SAP, however, which makes it difficult to link Treg numbers to autoantibody levels in our SAP cohort.

The limitation of the present study is a lack of data on the functional capabilities of Tregs in our cohorts of switched SAP and non-switched SAP. Limitations in blood sampling and cell quantities required for suppression assays made it unfeasible to assess the functional capabilities of Tregs in this prospective setting. Several studies reported on defects in Treg function in RA patients [11,14,35] while other studies could not confirm this observation [9,12,36]. Whether Treg function is affected in SAP during RA development remains therefore a topic for future studies. In our study circulating Tregs were assessed. This might be considered another limitation as synovial Tregs are important for suppressing ongoing joint inflammation.

In conclusion, we report similar numbers of peripheral CD4^+^CD25^+^FoxP3^+^ regulatory T-cells in HC, SAP and RA, although a trend towards reduced percentages of peripheral CD4^+^CD25^+^FoxP3^+^ regulatory T-cells in RA patients compared to HC was observed. Also, no differences in bonafide Treg subsets were observed. SAP did not show decreased numbers of peripheral CD4^+^CD25^+^FoxP3^+^ Tregs but did show an increase of CD45RA^-^FoxP3^low^ non-Treg cells (Fr III) compared to HC. When comparing within SAP, we found no significant differences in Treg frequencies between switched and non-switched SAP at inclusion. In addition, Treg numbers did not change in the 6 months prior to arthritis diagnosis. Hence, there is presumably no contribution of altered Treg numbers leading to the loss in suppression of autoimmunity in RA pathology. A broad IgG and IgA ACPA response in serum is thus more indicative as a biomarker for RA development in SAP than altered Treg numbers.

## Supporting Information

S1 Fig (A) IgG anti-CCP2 levels (n = 11) and (B) RF levels (n = 10) in switched SAP of whom data was available at the time of RA diagnosis and 6 months before switch to RA. Paired T-test was used to compare groups. No significant differences were observed. (C) Correlation between IgG anti-CCP2 levels and absolute numbers of Fr III in SAP and (D) correlation between absolute numbers of Fr III and number of peptides recognized (IgG) by SAP. Spearman rank test was used to assess correlations. No correlations were observed.
(TIF)

## References

[1] Klareskog L, Ronnelid J, Lundberg K, Padyukov L, Alfredsson L. Immunity to citrullinated proteins in rheumatoid arthritis. Annu Rev Immunol. 2008;26: 651–675. 10.1146/annurev.immunol.26.021607.090244 18173373

[2] Willemze A, Trouw LA, Toes RE, Huizinga TW. The influence of ACPA status and characteristics on the course of RA. Nat Rev Rheumatol. 2012;8: 144–152. 10.1038/nrrheum.2011.204 22293763

[3] Nielen MM, van Schaardenburg D, Reesink HW, van de Stadt RJ, van der Horst-Bruinsma IE, de Koning MH, et al Specific autoantibodies precede the symptoms of rheumatoid arthritis: a study of serial measurements in blood donors. Arthritis Rheum. 2004;50: 380–386. 14872479 10.1002/art.20018

[4] Shi J, van de Stadt LA, Levarht EW, Huizinga TW, Hamann D, van Schaardenburg D, et al Anti-carbamylated protein (anti-CarP) antibodies precede the onset of rheumatoid arthritis. Ann Rheum Dis. 2014;73: 780–783. 10.1136/annrheumdis-2013-204154 24336334

[5] Kokkonen H, Soderstrom I, Rocklov J, Hallmans G, Lejon K, Rantapaa Dahlqvist S. Up-regulation of cytokines and chemokines predates the onset of rheumatoid arthritis. Arthritis Rheum. 2010;62: 383–391. 10.1002/art.27186 20112361

[6] Sakaguchi S. Naturally arising CD4+ regulatory t cells for immunologic self-tolerance and negative control of immune responses. Annu Rev Immunol. 2004;22: 531–562. 15032588 10.1146/annurev.immunol.21.120601.141122

[7] Miyara M, Gorochov G, Ehrenstein M, Musset L, Sakaguchi S, Amoura Z. Human FoxP3+ regulatory T cells in systemic autoimmune diseases. Autoimmun Rev. 2011;10: 744–755. 10.1016/j.autrev.2011.05.004 21621000

[8] Lawson CA, Brown AK, Bejarano V, Douglas SH, Burgoyne CH, Greenstein AS, et al Early rheumatoid arthritis is associated with a deficit in the CD4+CD25high regulatory T cell population in peripheral blood. Rheumatology (Oxford). 2006;45: 1210–1217. 16571607 10.1093/rheumatology/kel089

[9] Samson M, Audia S, Janikashvili N, Ciudad M, Trad M, Fraszczak J, et al Brief report: inhibition of interleukin-6 function corrects Th17/Treg cell imbalance in patients with rheumatoid arthritis. Arthritis Rheum. 2012;64: 2499–2503. 10.1002/art.34477 22488116

[10] Cao D, Malmstrom V, Baecher-Allan C, Hafler D, Klareskog L, Trollmo C. Isolation and functional characterization of regulatory CD25brightCD4+ T cells from the target organ of patients with rheumatoid arthritis. Eur J Immunol. 2003;33: 215–223. 12594850 10.1002/immu.200390024

[11] Ehrenstein MR, Evans JG, Singh A, Moore S, Warnes G, Isenberg DA, et al Compromised function of regulatory T cells in rheumatoid arthritis and reversal by anti-TNFalpha therapy. J Exp Med. 2004;200: 277–285. 15280421 10.1084/jem.20040165 PMC2211983

[12] Liu MF, Wang CR, Fung LL, Lin LH, Tsai CN. The presence of cytokine-suppressive CD4+CD25+ T cells in the peripheral blood and synovial fluid of patients with rheumatoid arthritis. Scand J Immunol. 2005;62: 312–317. 16179019 10.1111/j.1365-3083.2005.01656.x

[13] Mottonen M, Heikkinen J, Mustonen L, Isomaki P, Luukkainen R, Lassila O. CD4+ CD25+ T cells with the phenotypic and functional characteristics of regulatory T cells are enriched in the synovial fluid of patients with rheumatoid arthritis. Clin Exp Immunol. 2005;140: 360–367. 15807863 10.1111/j.1365-2249.2005.02754.x PMC1809357

[14] Nie H, Zheng Y, Li R, Guo TB, He D, Fang L, et al Phosphorylation of FOXP3 controls regulatory T cell function and is inhibited by TNF-alpha in rheumatoid arthritis. Nat Med. 2013;19: 322–328. 10.1038/nm.3085 23396208

[15] van Amelsfort JM, Jacobs KM, Bijlsma JW, Lafeber FP, Taams LS. CD4(+)CD25(+) regulatory T cells in rheumatoid arthritis: differences in the presence, phenotype, and function between peripheral blood and synovial fluid. Arthritis Rheum. 2004;50: 2775–2785. 15457445 10.1002/art.20499

[16] Miyara M, Yoshioka Y, Kitoh A, Shima T, Wing K, Niwa A, et al Functional delineation and differentiation dynamics of human CD4+ T cells expressing the FoxP3 transcription factor. Immunity. 2009;30: 899–911. 10.1016/j.immuni.2009.03.019 19464196

[17] van de Stadt LA, Witte BI, Bos WH, van Schaardenburg D. A prediction rule for the development of arthritis in seropositive arthralgia patients. Ann Rheum Dis. 2013;72: 1920–1926. 10.1136/annrheumdis-2012-202127 23178208

[18] van de Stadt LA, van der Horst AR, de Koning MH, Bos WH, Wolbink GJ, van de Stadt RJ, et al The extent of the anti-citrullinated protein antibody repertoire is associated with arthritis development in patients with seropositive arthralgia. Ann Rheum Dis. 2011;70: 128–133. 10.1136/ard.2010.132662 21062853

[19] Demoruelle MK, Deane KD, Holers VM. When and where does inflammation begin in rheumatoid arthritis? Curr Opin Rheumatol. 2014;26: 64–71. 10.1097/BOR.0000000000000017 24247116 PMC4033623

[20] Kokkonen H, Mullazehi M, Berglin E, Hallmans G, Wadell G, Ronnelid J, et al Antibodies of IgG, IgA and IgM isotypes against cyclic citrullinated peptide precede the development of rheumatoid arthritis. Arthritis Res Ther. 2011;13: R13 10.1186/ar3237 21291540 PMC3241357

[21] van der Geest KS, Abdulahad WH, Tete SM, Lorencetti PG, Horst G, Bos NA, et al Aging disturbs the balance between effector and regulatory CD4+ T cells. Exp Gerontol. 2014;60: 190–196. 10.1016/j.exger.2014.11.005 25449852

[22] Janssen KM, de Smit MJ, Brouwer E, de Kok FA, Kraan J, Altenburg J, et al Rheumatoid arthritis-associated autoantibodies in non-rheumatoid arthritis patients with mucosal inflammation: a case-control study. Arthritis Res Ther. 2015;17: 174-015-0690-6. 10.1186/s13075-015-0690-6 PMC4496865 26155788

[23] Smolen JS, Aletaha D. Rheumatoid arthritis therapy reappraisal: strategies, opportunities and challenges. Nat Rev Rheumatol. 2015;11: 276–289. 10.1038/nrrheum.2015.8 25687177

[24] Arend WP, Firestein GS. Pre-rheumatoid arthritis: predisposition and transition to clinical synovitis. Nat Rev Rheumatol. 2012;8: 573–586. 10.1038/nrrheum.2012.134 22907289

[25] Karlson EW, van Schaardenburg D, van der Helm-van Mil AH. Strategies to predict rheumatoid arthritis development in at-risk populations. Rheumatology (Oxford). 2016;55: 6–15. 25096602 10.1093/rheumatology/keu287 PMC4676903

[26] Wang W, Shao S, Jiao Z, Guo M, Xu H, Wang S. The Th17/Treg imbalance and cytokine environment in peripheral blood of patients with rheumatoid arthritis. Rheumatol Int. 2012;32: 887–893. 10.1007/s00296-010-1710-0 21221592

[27] Ponchel F, Goeb V, Parmar R, El-Sherbiny Y, Boissinot M, El Jawhari J, et al An immunological biomarker to predict MTX response in early RA. Ann Rheum Dis. 2014;73: 2047–2053. 10.1136/annrheumdis-2013-203566 23989988

[28] Chalan P, Kroesen BJ, van der Geest KS, Huitema MG, Abdulahad WH, Bijzet J, et al Circulating CD4+CD161+ T lymphocytes are increased in seropositive arthralgia patients but decreased in patients with newly diagnosed rheumatoid arthritis. PLoS One. 2013;8: e79370 10.1371/journal.pone.0079370 24223933 PMC3815125

[29] van de Sande MG, de Hair MJ, van der Leij C, Klarenbeek PL, Bos WH, Smith MD, et al Different stages of rheumatoid arthritis: features of the synovium in the preclinical phase. Ann Rheum Dis. 2011;70: 772–777. 10.1136/ard.2010.139527 21177292

[30] Matsuki F, Saegusa J, Miyamoto Y, Misaki K, Kumagai S, Morinobu A. CD45RA-Foxp3(high) activated/effector regulatory T cells in the CCR7 + CD45RA-CD27 + CD28+central memory subset are decreased in peripheral blood from patients with rheumatoid arthritis. Biochem Biophys Res Commun. 2013;438: 778–783. 10.1016/j.bbrc.2013.05.120 23747721

[31] Hunt L, Hensor EM, Nam J, Burska AN, Parmar R, Emery P, et al T cell subsets: an immunological biomarker to predict progression to clinical arthritis in ACPA-positive individuals. Annals of the Rheumatic Diseases. 2015 [Published on 1 Dec 2015] 10.1136/annrheumdis-2015-207991 PMC5036223 27613874

[32] Brink M, Hansson M, Mathsson L, Jakobsson PJ, Holmdahl R, Hallmans G, et al Multiplex analyses of antibodies against citrullinated peptides in individuals prior to development of rheumatoid arthritis. Arthritis Rheum. 2013;65: 899–910. 10.1002/art.37835 23310951

[33] Jang E, Cho WS, Cho ML, Park HJ, Oh HJ, Kang SM, et al Foxp3+ regulatory T cells control humoral autoimmunity by suppressing the development of long-lived plasma cells. J Immunol. 2011;186: 1546–1553. 10.4049/jimmunol.1002942 21209284

[34] Morgan ME, Sutmuller RP, Witteveen HJ, van Duivenvoorde LM, Zanelli E, Melief CJ, et al CD25+ cell depletion hastens the onset of severe disease in collagen-induced arthritis. Arthritis Rheum. 2003;48: 1452–1460. 12746920 10.1002/art.11063

[35] Alvarado-Sanchez B, Hernandez-Castro B, Portales-Perez D, Baranda L, Layseca-Espinosa E, Abud-Mendoza C, et al Regulatory T cells in patients with systemic lupus erythematosus. J Autoimmun. 2006;27: 110–118. 16890406 10.1016/j.jaut.2006.06.005

[36] Han GM, O'Neil-Andersen NJ, Zurier RB, Lawrence DA. CD4+CD25high T cell numbers are enriched in the peripheral blood of patients with rheumatoid arthritis. Cell Immunol. 2008;253: 92–101. 10.1016/j.cellimm.2008.05.007 18649874 PMC2585376

