# Supplementary figures and images for: Regulatory CD4+ T-Cell Subsets and Anti-Citrullinated Protein Antibody Repertoire: Potential Biomarkers for Arthritis Development in Seropositive Arthralgia Patients?
Source: PLoS One. 2016 Sep 1;11(9):e0162101. doi: 10.1371/journal.pone.0162101 (PMC5008772; doi:10.1371/journal.pone.0162101)

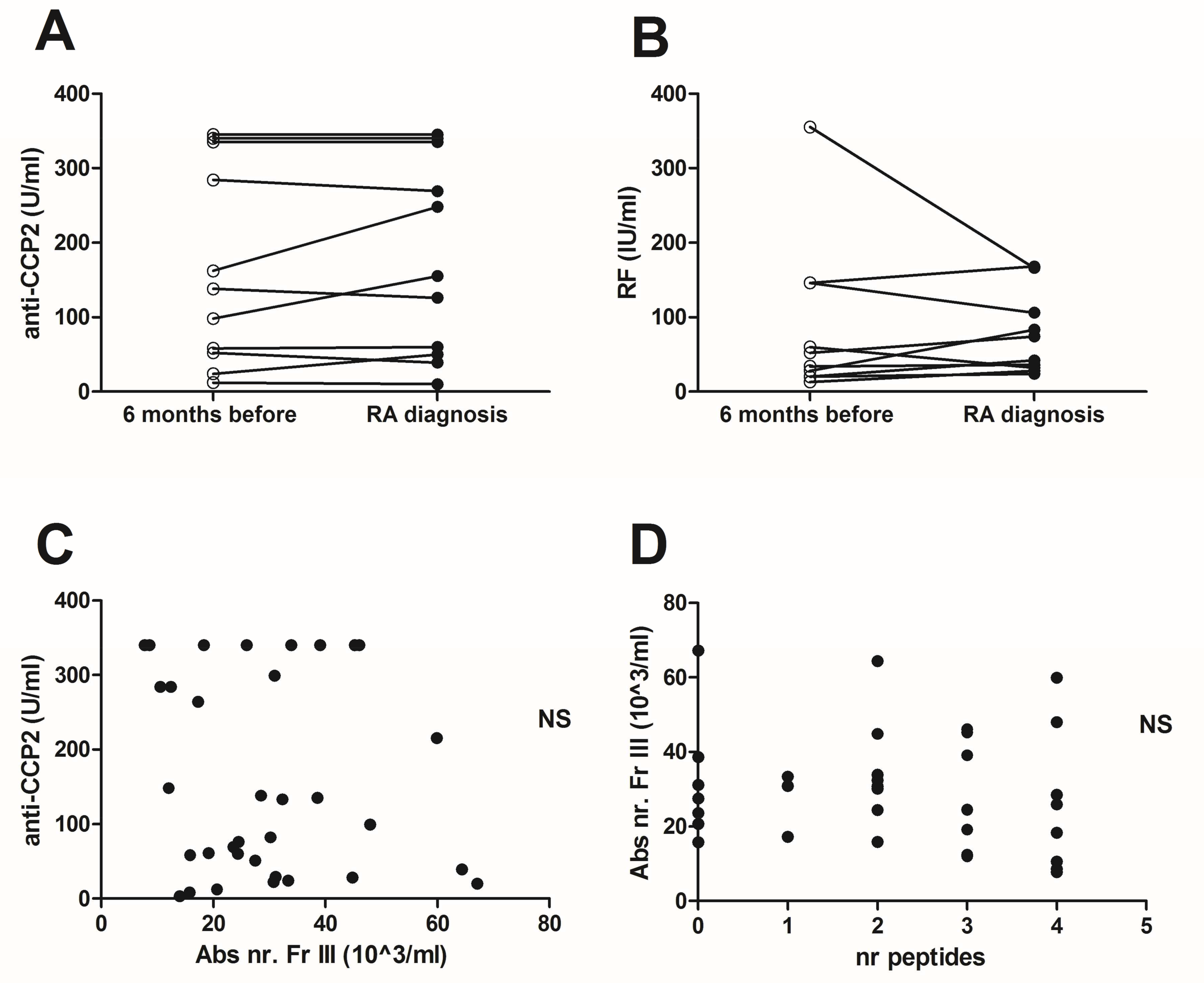

Supplement: S1 Fig — (A) IgG anti-CCP2 levels (n = 11) and (B) RF levels (n = 10) in switched SAP of whom data was available at the time of RA diagnosis and 6 months before switch to RA. Paired T-test was used to compare groups. No significant differences were observed. (C) Correlation between IgG anti-CCP2 levels and absolute numbers of Fr III in SAP and (D) correlation between absolute numbers of Fr III and number of peptides recognized (IgG) by SAP. Spearman rank test was used to assess correlations. No correlations were observed. (TIF) [file pone.0162101.s001.tif]
